# Activation of the Supplementary Motor Areas Enhances Spinal Reciprocal Inhibition in Healthy Individuals

**DOI:** 10.3390/brainsci10090587

**Published:** 2020-08-24

**Authors:** Ryo Hirabayashi, Sho Kojima, Mutsuaki Edama, Hideaki Onishi

**Affiliations:** Institute for Human Movement and Medical Sciences, Niigata University of Health and Welfare, Niigata 950-3198, Japan; kojima@nuhw.ac.jp (S.K.); edama@nuhw.ac.jp (M.E.); onishi@nuhw.ac.jp (H.O.)

**Keywords:** H-reflex, M wave, electromyography, transcranial direct current stimulation, RI enhancement

## Abstract

The supplementary motor area (SMA) may modulate spinal reciprocal inhibition (RI) because the descending input from the SMA is coupled to interneurons in the spinal cord via the reticulospinal tract. Our study aimed to verify whether the anodal transcranial direct current stimulation (anodal-tDCS) of the SMA enhances RI. Two tDCS conditions were used: the anodal stimulation (anodal-tDCS) and sham stimulation (sham-tDCS) conditions. To measure RI, there were two conditions: one with the test stimulus (alone) and the other with the conditioning-test stimulation intervals (CTIs), including 2 ms and 20 ms. RI was calculated at multiple time points: before the tDCS intervention (Pre); at 5 (Int 5) and 10 min; and immediately after (Post 0); and at 5, 10 (Post 10), 15, and 20 min after the intervention. In anodal-tDCS, the amplitude values of H-reflex were significantly reduced for a CTI of 2 ms at Int 5 to Post 0, and a CTI of 20 ms at Int 5 to Pot 10 compared with Pre. Stimulation of the SMA with anodal-tDCS for 15 min activated inhibitory interneurons in RIs by descending input from the reticulospinal tract via cortico–reticulospinal projections. The results showed that 15 min of anodal-tDCS in the SMA enhanced and sustained RI in healthy individuals.

## 1. Introduction

Dysfunction of neurons of the upper motor system [1,2,3], spinal cord injury [4], and aging [5,6,7,8,9,10] disrupt smooth joint movement owing to excessive simultaneous activation (ESA) of antagonist muscles, thus decreasing exercise performance. ESA in the lower limbs degrades gait functions and increases falling risk [11,12]. Further, during joint exercises based on repetitive and quick actions, ESA causes inhibition of smooth joint movement and triggers fatigue in the agonist muscle [13]. The mechanism underlying these ESAs is the repression of interneurons of reciprocal inhibition (RI) by Renshaw cells [1,2,3].

RI is an essential function for normal joint movement, balance and gait, and involves three inhibitory pathways. These pathways include disynaptic Ia RI (DRI) that directly links to spinal anterior horn cells (SAHC) of antagonist muscles [14,15] and presynaptic short (D1 inhibition) and long (D2 inhibition) latency inhibition pathways mediated by primary afferent depolarization (PAD) interneurons [14]. These RI pathways enable and control coordinated movement by inhibiting excessive contraction of antagonist muscles [5,9,13].

Many studies report the enhancement of RI aimed at inhibiting excessive simultaneous activity and improving coordinated movement and gait [16,17,18,19,20,21,22,23,24,25,26]. In particular, to enhance RI, peripheral patterned electrical stimulation (PES) [18,20,24,26,27] and repetitive passive movement (RPM) [16,17] are more effective than brain stimulation. A groundbreaking report shows a synergistic impact after combined brain and peripheral stimulation [24,26]. In previous studies, primary motor area (M1) in the brain was activated using transcranial direct current stimulation (tDCS) [24]. Alternatively, some studies used intermittent theta-burst transcranial magnetic stimulation (iTBS) [26]. However, in brain stimulation alone, only enhancement of DRI during intervention was observed in tDCS and aftereffects were not present [19,21,24]. ITBS, which is capable of local activity, enhanced D1 inhibition only immediately after intervention; however, no sustained aftereffects of DRI or D1 inhibition during or after the intervention [26]. The activity of M1 by tDCS or iTBS alone did not exhibit adequate aftereffects.

We focused on the supplementary motor area (SMA), a brain region with the potential to enhance RI. The SMA is heavily involved in planning of both simple and complex motor behavior and in behavior sequencing, learning, and motor control [28,29,30,31]. Stimulating the activity of the SMA by anodal-tDCS and TMS improves gait, balance, and coordinated movement [32,33,34]. SMAs display an abundance of cortical reticular projection origins and increase descending input from the reticulospinal tract via cortico–reticulospinal projections [35,36]. The reticulospinal tract has a large number of nerve endings in the gray matter of the spinal cord and projects to intervening cells in this region [37]. We hypothesized that the activity of the reticular spinal tract modulates RI because it is involved in inhibiting muscle tone and improving gait and motor control [38,39].

The purpose of this study was to examine whether RI is enhanced by anodal-tDCS of the SMA.

## 2. Materials and Methods

To determine whether anodal-tDCS to the SMA enhances RI, we intervened in the SMA using tDCS for 15 min and examined the amount of RI (DRI and D1 inhibition) in the right soleus (Sol) muscle H-reflex.

### 2.1. Study Participants

We recruited 20 healthy male adults with an average age of 21.4 ± 0.5 years, a height of 170.9 ± 6.6 cm, and bodyweight of 62.5 ± 6.5 kg. All participants provided written informed consent to participate. This study was approved by the University Ethics Committee (18310—191101). All experiments were conducted in accordance with the University ethical standards and the 1964 Helsinki Declaration and its later amendments.

### 2.2. Measurement of Limb Position

**Figure 1:**
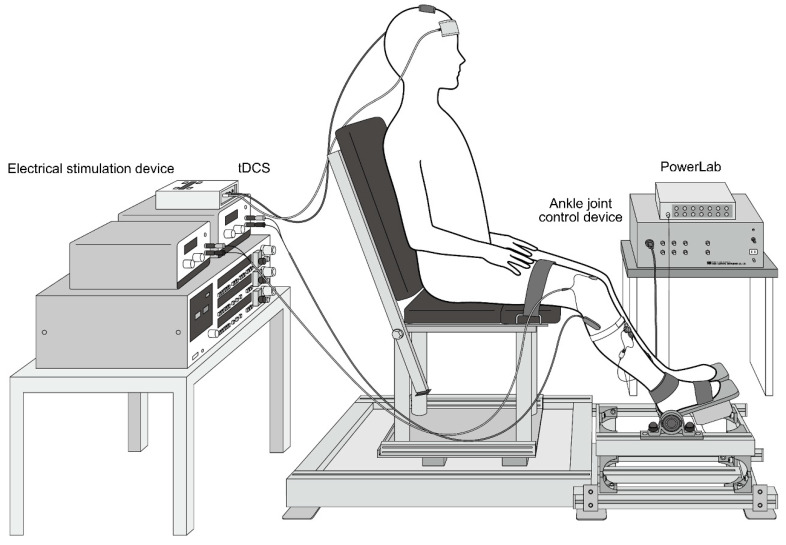
Limb position.

As in our previous studies [16,17], the position of the right lower limb was measured at the hip (100°), knee (120°), and ankle joints (110°). Thigh and foot were fixed to a seat and footplate, respectively (Takei Scientific Instruments, Niigata, Japan) (Figure 1), to maintain participants’ posture during the experiment.

### 2.3. Electromyography (EMG)

EMG was performed using Ag/AgCl electrodes (Blue Sensor, METS, Tokyo, Japan) with a distance of 20 mm between the electrodes. Electrodes were positioned in the tibialis anterior (TA) and Sol [40]; ground electrodes were placed between electrical stimulation electrodes and surface EMG electrodes on the TA muscle [41,42]. EMG activity was filtered using a 10–1000 Hz bandpass filter and amplified 100× (FA-DL-720-140; 4Assist, Tokyo, Japan) before being digitally stored on a personal computer for offline analyses. The sampling rate was 10 kHz. PowerLab 8/30 and LabChart 7 (both AD Instruments, Colorado Springs, CO, USA) were used for data analyses.

### 2.4. tDCS

tDCS was delivered by a direct current stimulator (Eldith, neuroConn GmbH, Ilmenau, Germany). A pair of sponge electrodes (5 × 7 cm^2^, 35 cm^2^) soaked in saline were employed. The anodal electrode was placed in the SMA, and the cathodal electrode was placed on the right orbit. The sagittal midline was 3 cm anterior to the parietal lobe [43,44,45,46]. This placement was consistent with the International system 10–20. Two conditions were established for the tDCS intervention: the anodal stimulation condition (anodal-tDCS) and the sham stimulation condition (sham-tDCS). For anodal-tDCS, a 1 mA current was set (with current density = 0.028 mA/cm^2^) [24] and duration of current application was set to 15 min, with fade in and fade out times of 10 s. This same stimulation method was applied for sham-tDCS except for the duration of current application which was limited to 15 s with a fade in and fade out time of 10 s.

### 2.5. Electrical Stimulation

Nerve stimulation was induced by 1 ms square wave pulses using an SS-104J isolator (Nihon Kohden, Tokyo, Japan) and a SEN-8203 electrical stimulator (Nihon Kohden). Nerve stimulation was performed using the same technique as that used in our previous study [16,17], with the test stimulus administered to the tibial nerve and the conditional stimulus to the common peroneal nerve.

### 2.6. Reciprocal Inhibition

RI was measured in the same manner as done in previous studies [14,16,26,47,48]. Sol H-reflex amplitude values were measured after conditioning and test stimuli. The intensity of the conditioning stimulus was set to the M-wave threshold (stimulus intensity evoking <100 μV) of TA [14,17,26]. Conditioning stimuli were carefully placed so as not to cause peroneal muscle contraction [26,47,48]. Because the degree of RI varied with the magnitude of H-reflex [49], the intensity of test stimuli was set to induce an H-reflex of 15–25% of the maximum amplitude value of the Sol M wave (Mmax). The three stimulus conditions were a conditioning-test stimulus interval (CTI) of 2 ms or 20 ms and a test stimulus without a conditioning stimulus (single). A CTI of 2 ms reportedly produces the greatest enhancement of DRI [14,50], and that of 20 ms reportedly produces the greatest enhancement of D1 inhibition [14]. The number of stimulations administered was randomly set to 36 (three stimulation conditions × 12 sets). Stimulation frequency was set at 0.3 Hz. At this frequency, H-reflex was measured after at least three stimuli because H-reflex stabilizes after the third stimulus [51].

### 2.7. Experimental Protocol

**Figure 2:**
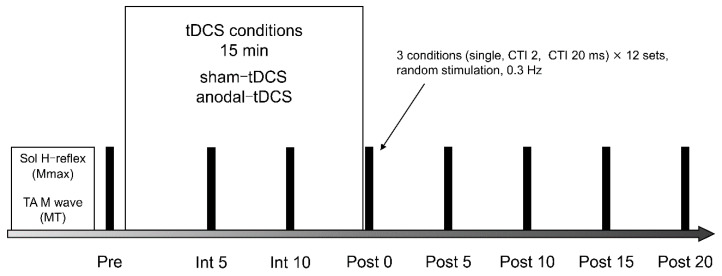
Experiment protocol.

The detailed procedure followed is illustrated in Figure 2. The intensity of conditioned and test stimuli was set before RI measurements. RI was assessed before stimulation (Pre); at 5 (Int 5) and 10 (Int 10) min during; immediately after (Post 0); and at 5 (Post 5), 10 (Post 10), 15 (Post 15), and 20 (Post 20) min after the tDCS intervention. Intervention for tDCS conditioning was 15 min, and two conditions (sham-tDCS, anodal-tDCS) were performed randomly. Interventions were implemented at intervals of ≥1 week.

The two tDCS conditions, i.e., the anodal-tDCS condition (anodal-tDCS) and the sham-tDCS condition (sham-tDCS), were performed randomly in the SMA for 15 min. Each tDCS intervention was conducted at an interval of ≥1 week. The Mmax of the Sol and the MT of the TA were measured before the RI measurements. RI was measured under three conditions.

MT, motor threshold; SMA, supplementary motor area; tDCS, transcranial direct current stimulation

### 2.8. Statistical Analyses

For Sol and TA, H-reflex and M-wave amplitude values were measured by averaging peak-to-peak values of waveform amplitude of each stimulation condition. RI was calculated as a percentage (%) by dividing Sol H-reflex amplitude value by Mmax amplitude value. Percent notation was calculated by dividing the H-reflex amplitude value of the conditioned stimulus by H-reflex amplitude value of the test stimulus alone ([conditioned H-reflex amplitude value/test H-reflex amplitude value] × 100). Background EMG (Sol), Mmax amplitude (Sol), and M-wave amplitude (TA) values during the experiments were analyzed as reference data. Effects of tDCS condition, stimulation condition, and measurement time was analyzed by repeated-measures three-way analysis of variance (ANOVA). As a post-hoc analysis, the single and two stimulation conditions, for each tDCS intervention, were compared using paired *t*-tests with Bonferroni correction. Comparisons of measurement times for each tDCS condition were performed using paired *t*-tests with Bonferroni correction. Statistical significance was considered at *p* < 0.05.

## 3. Results

The Sol muscle background EMG, Mmax amplitude values of the Sol muscle, and the M-wave amplitude values of the TA muscle are presented in Table 1a–c.

Three-way ANOVA (tDCS condition, stimulation condition and measurement time) demonstrated a primary effect of the tDCS conditioning (F(1, 19) = 4.717, *p* = 0.043, partial η^2^ = 0.199) and a primary effect of the stimulus condition (F(2, 38) = 185.152, *p* < 0.001, partial η^2^ = 0.907) but not of measurement time (F(7, 133) = 1.387, *p* = 0.216, partial η^2^ = 0.068). In addition, there was a significant interaction among the three factors (F(14, 266) = 2.221, *p* = 0.007, partial η^2^ = 0.105). Among the measurement times, no significant differences were observed in Sol H-reflex amplitude values for a single condition (Table 2). Thus, after conditioning stimuli, changes in Sol H-reflex amplitude values were independent of the test stimulus intensity.

H-reflex amplitude values after single or two stimuli were compared, and at each time interval, H-reflex amplitude decreased significantly for CTIs of 2 and 20 ms after two stimuli compared with that after a single stimulus *(p* < 0.001, Table 2). DRI and D1 inhibition were observed under all conditions.

**Figure 3:**
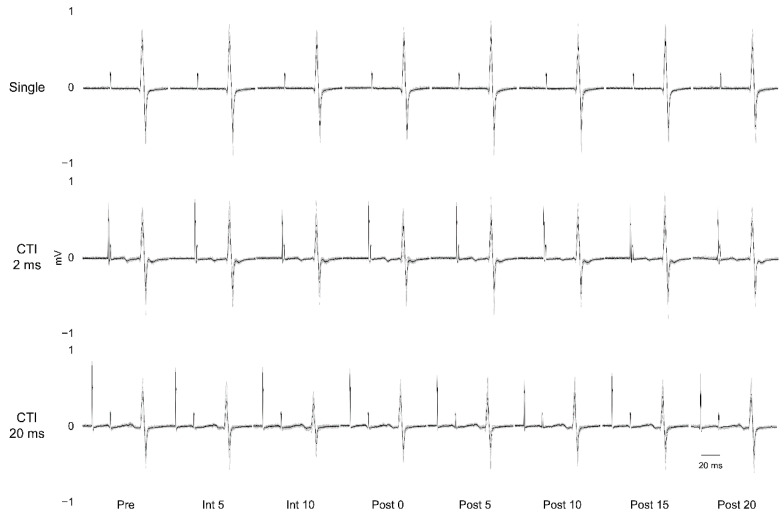
Tracing raw data-Sol muscle. The figure depicts a representative raw data-trace of a subject during anodal-tDCS. Stimulation conditions: single, CTI: 2 ms (DRI), and CTI: 20 ms (D1 inhibition), and waveforms of Sol H-reflex: 12. Bold black lines are averages of the 12 waveforms. The horizontal axis is the measurement time of RI.

**Figure 4:**
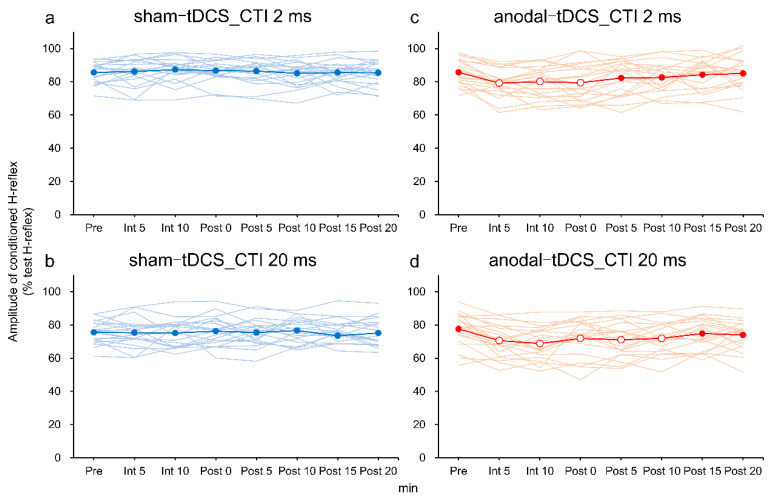
Comparison of the temporal changes in RI. (**a**,**b**) Sham-tDCS; (**c**,**d**) anodal-tDCS; (**a**,**c**) CTI of 2 ms; and (**b**,**d**) CTI of 20 ms. The thin solid lines show changes over time for 20 subjects, and the thick solid line indicates mean values. The vertical axis is the amplitude of the conditioning H-reflex/amplitude of the test H-reflex × 100. The horizontal axis is the measurement time of RI. Pre values were compared with those at different measurement times using a paired *t*-test with Bonferroni correction. Values indicated by filled symbols did not show significant differences compared with the Pre values. The open symbols indicate values significantly different from the Pre values *(p* < 0.05). CTI, conditioning stimulation–test stimulation interval; tDCS, transcranial direct current stimulation.

Changes in the H-reflex amplitude values at CTIs of 2 and 20 ms were compared with the Pre values (Figure 3 and Figure 4). The sham-tDCS condition at CTIs of 2 ms and 20 ms did not produce any significant change in H-reflex amplitude values compared with Pre values. Conversely, anodal-tDCS caused significant reduction in H-reflex amplitude values at Int 5 (*p* < 0.001), Int 10 (*p* = 0.025), and Post 0 (*p* = 0.034) compared with the Pre values. Similarly, anodal-tDCS led to significantly reduced H-reflex amplitude value with CTI of 20 ms at Int 5 (*p* = 0.003), Int 10 (*p* < 0.001), Post 0 (*p* = 0.018), Post 5 (*p* = 0.006), and Post 10 (*p* = 0.041).

## 4. Discussion

This study is the first to report that SMA activity enhances RI. Intervention in the SMA via anodal-tDCD for 15 min upregulated DRI from Int 5 to Post 0 and also upregulated the D1 inhibition from Int 5 to Post 10.

The results in the current study demonstrated that in comparison with single stimuli, H-reflex amplitude values were significantly reduced at CTIs of 2 and 20 ms for all tDCS conditions. This finding is indicative of the presence of DRI and D1 inhibition [14,15].

Previous studies of brain stimulation for RI enhancement report M1 stimulation [19,20,21,24,26]. An intervention of anodal-tDCS in M1 shows that DRI produces no aftereffects, whereas there was an increase in inhibition during the intervention; the authors reported no changes in D1 inhibition [19,20,21,24,26]. These aftereffects might be explained by the hypothesis that tDCS applied to the lower limb region of M1 causes concomitant activation of the TA and Sol regions. As the activity is similar to that of M1 during co-contraction, it is considered that RI was inhibited and aftereffects were not observed [24]. Conversely, iTBS, which activates more localized brain regions than tDCS, enhanced D1 inhibition immediately after the intervention, whereas DRI remained unchanged [26]. Compared with these previous studies, the current study found that anodal-tDCS of the SMA is an efficacious method of brain stimulation to enhance RI. Both DRI and D1 inhibition yielded aftereffects during and immediately after the intervention; in addition, D1 inhibition led to aftereffects until Post 10.

The SMA is the origin of the cortico–reticulospinal projection and mobilizes much of the reticulospinal tract via this projection [35,36]. The reticulospinal tract, via the cortico–reticulospinal projection, displays a large number of nerve endings in the spinal gray matters and projects intervening inhibitory cells in this region [37]. These cells contain many inhibitory interneurons (Ia inhibitory and PAD interneurons) that support DRI and D1 inhibition. Thus, downward input provided by SMA activity could activate inhibitory interneurons and subsequently lead to enhancement of RI. The activity of the SMA improves gait, balance and coordinated movement [32,33,34], and RI plays an important role in these functions. Therefore, it was suggested that SMA and RI is involved in an important regulatory pathway.

The current study had an experimental design that required rapid measurement of RI before and after the tDCS conditioning. Thus, we could not evaluate the excitability of SAHCs. This parameter would have been measured by H/M recruitment curves. Moreover, a few previously performed studies [18,24,26,27] that calculated RI, had also calculated Hmax using stimulus intensity of the Sol H-reflex normalized to Mmax. The current study used the same method to quickly calculate RI and assess the aftereffects of tDCS in real-time.

### Clinical Application

The SMA activity explored in this study suggests an efficacious therapy that could be used as adjuvant therapy for the revival of associated motor functions following central nervous system injury. This study shows an effective brain-stimulation intervention for RI enhancement in healthy individuals. Previous brain stimulation methods did not report an enhancement of both DRI and D1 inhibition. Further, D1 inhibition resulted in aftereffects persisting up to 10 min after the intervention. Prolonging the duration of aftereffects is an effective adjuvant therapy for rehabilitation. Future studies should examine a combination of brain stimulation (targeting SMA) with RPM or PES because the combination of brain and peripheral stimulation is expected to prolong the aftereffect. In addition, next steps should include validation of the long-term effects of this therapy, including improvement of the functional abnormalities and dysregulated motor function in patients.

## 5. Conclusions

The present study examined whether intervention with tDCS of the SMA would contribute to RI enhancement. The results showed that DRI and D1 inhibition were both enhanced during and immediately after the intervention. In addition, D1 inhibition persisted up to 10 min after the intervention. This study is the first to report that anodal-tDCS intervention in the SMA contributes to RI enhancement in healthy individuals.

## Figures and Tables

**Table 1:** The Sol muscle background EMG, Mmax amplitude values of the Sol muscle, and the M-wave amplitude values of the TA muscle.

**a.** Background electromyography (EMG) (Sol)								
	**Pre**	**Int 5**	**Int 10**	**Post 0**	**Post 5**	**Post 10**	**Post 15**	**Post 20**
Sham-tDCS	2.9 ± 0.1	2.9 ± 0.1	2.9 ± 0.1	2.9 ± 0.1	2.9 ± 0.1	3.0 ± 0.1	2.9 ± 0.1	2.8 ± 0.1
Anodal-tDCS	3.1 ± 0.3	3.1 ± 0.2	3.4 ± 0.3	3.4 ± 0.3	3.2 ± 0.2	3.1 ± 0.3	3.1 ± 0.2	3.3 ± 0.3
**b.** Mmax amplitude values (Sol)								
	**Sham-tDCS**				**Anodal-tDCS**			
	10.96 ± 0.81				11.58 ± 0.92			
**c.** M-wave amplitude values (TA)								
	**Pre**	**Int 5**	**Int 10**	**Post 0**	**Post 5**	**Post 10**	**Post 15**	**Post 20**
Sham-tDCS	89.0 ± 1.6	90.1 ± 1.9	88.6 ± 1.3	87.7 ± 2.0	88.6 ± 1.3	91.0 ± 1.8	87.5 ± 1.8	87.9 ± 1.9
Anodal-tDCS	88.1 ± 3.0	88.4 ± 3.6	89.4 ± 3.1	90.2 ± 2.1	88.0 ± 3.0	88.2 ± 3.5	85.7 ± 5.2	86.9 ± 3.6

**a.** The data represents values as Mean ± SE. Sol background EMG (μV), EMG was measured 30–50 ms before the test stimulus). **b.** The data represents values as Mean ± SE (mV). **c.** Data are reported as mean ± SE (μV).

**Table 2:** H-reflex amplitude (% Mmax).

		Pre	Int 5	Int 10	Post 0	Post 5	Post 10	Post 15	Post 20
Sham-tDCS	Single	20.0 ± 0.3	19.7 ± 0.3	20.4 ± 0.3	20.3 ± 0.2	20.2 ± 0.3	20.7 ± 0.3	20.5 ± 0.3	20.4 ± 0.4
	CTI 2 ms	17.1 ± 0.4 ‡	17.0 ± 0.4 ‡	17.8 ± 0.4 ‡	17.6 ± 0.4 ‡	17.5 ± 0.4 ‡	17.6 ± 0.4 ‡	17.5 ± 0.4 ‡	17.4 ± 0.5 ‡
	CTI 20 ms	15.2 ± 0.4 ‡	14.8 ± 0.4 ‡	15.3 ± 0.4 ‡	15.4 ± 0.4 ‡	15.3 ± 0.4 ‡	15.9 ± 0.4 ‡	15.7 ± 0.4 ‡	15.3 ± 0.4 ‡
Anodal-tDCS	Single	20.4 ± 0.4	20.6 ± 0.4	20.1 ± 0.3	20.1 ± 0.3	20.2 ± 0.4	20.5 ± 0.3	20.4 ± 0.3	19.6 ± 0.3
	CTI 2 ms	17.1 ± 0.4 ‡	16.2 ± 0.4 ‡	16.0 ± 0.5 ‡	16.0 ± 0.6 ‡	16.6 ± 0.5 ‡	17.0 ± 0.5 ‡	17.1 ± 0.4 ‡	16.7 ± 0.5 ‡
	CTI 20 ms	15.4 ± 0.4 ‡	14.5 ± 0.5 ‡	13.8 ± 0.4 ‡	14.4 ± 0.6 ‡	14.3 ± 0.5 ‡	14.8 ± 0.5 ‡	15.2 ± 0.5 ‡	14.6 ± 0.5 ‡

The data represents values as Mean ± SE. The table depicts the results of every measurement time and for every tDCS condition. The Sol H-reflex amplitude values were calculated as mean ± SE of the peak-to-peak values of amplitude of each waveform as H-reflex/Mmax × 100. (Paired *t*-test with Bonferroni correction was performed; ‡ *p* < 0.001).
